# Down-regulation of tensin2 enhances tumorigenicity and is associated with a variety of cancers

**DOI:** 10.18632/oncotarget.9411

**Published:** 2016-05-17

**Authors:** Shiao-Ya Hong, Yi-Ping Shih, Peng Sun, Wang-Ju Hsieh, Wen-Chang Lin, Su Hao Lo

**Affiliations:** ^1^ Department of Biochemistry and Molecular Medicine, University of California-Davis, Sacramento, CA 95817, USA; ^2^ Institute of Biomedical Sciences, Academia Sinica, Taipei 115, Taiwan

**Keywords:** tensin, IRS1, Mek, focal adhesion, tumorigenesis

## Abstract

Tensin family members, including tensin2 (TNS2), are present as major components of the focal adhesions. The N-terminal end of TNS2 contains a C1 region (protein kinase C conserved region 1) that is not found in other tensin members. Three isoforms of TNS2 have been identified with previous reports describing the shortest V3 isoform as lacking the C1 region. Although TNS2 is known to regulate cell proliferation and migration, its role in tumorigenicity is controversial. By gain-of-function overexpression approaches, results supporting either promotion or reduction of cancer cell tumorigenicity were reported. Here we report that the complete V3 isoform also contains the C1 region and describe the expression patterns of the three human TNS2 isoforms. By loss-of-function approaches, we show that silencing of TNS2 up-regulates the activities of Akt, Mek, and IRS1, and increases tumorigenicities in A549 and Hela cells. Using public database analyses we found that TNS2 is down-regulated in head and neck, esophageal, breast, lung, liver, and colon cancer. In addition, patients with low TNS2 expression showed poor relapse-free survival rates for breast and lung cancers. These results strongly suggest a role of tensin2 in suppressing cell transformation and reduction of tumorigenicity.

## INTRODUCTION

Focal adhesions are transmembrane junctions linking the extracellular matrix to the cytoskeletons. They are critical structures in organizing the cytoskeleton network and transmitting signals that regulate cell adhesion, migration, proliferation, differentiation, and tissue development [1]. When the functions of focal adhesions go awry, they may lead to disease formation [1]. Tensin2 (TNS2) is a member of the tensin family that includes tensin1, tensin3, and cten [2]. All four tensin proteins localize to focal adhesions and contain the PTP (protein tyrosine phosphatase), SH2 (Src homology 2), and PTB (phosphotyrosine binding) domains, except that cten is shorter and does not share the PTP domain [2]. In addition, TNS2 features a C1 region (protein kinase C conserved region 1) that is not found in other tensin members. TNS2 is reported to regulate cell proliferation and migration [3, 4] and to be critical for cell to contract collagen gels [5]. The role of TNS2 in tumorigenesis is somewhat confusing. Over-expression of one tensin2 variant (V2, 1409aa) in BEL7402 and HepG2 reduced their colony formation activities [6]. On the other hand, over-expression of another TNS2 variant lacking the C1 region (1285aa) promoted BEL7402 cell proliferation, migration, and colony formation, as well as tumor growth in orthotopic liver xenograft [7]. In low serum condition, HEK293 cells transfected with TNS2 (V2, 1409aa) proliferated slower than control cells [3]. Whether these discrepancies are caused by different cell types and/or TNS2 variants are not clear.

The TNS2 knockout mice have not yet been reported. However, the role of TNS2 in mice has been suggested by an ICR-derived glomerulonephritis (ICGN) mouse model. TNS2 harboring an 8-nucleotide deletion that led to its early termination was believed to be the cause of the congenital nephrotic syndrome in the ICGN mice [8]. It was shown that the number of glomerular podocytes was significantly reduced, which might result in proteinuria that induced chronic kidney disease in the ICGN mice [9]. However, the phenotype of ICGN is dictated by mouse genetic backgrounds. By backcrossing and tracking the TNS2 mutant allele, renal phenotypes were only detected in homozygous TNS2 mutant mice in the BDA/2J or FBV/N strains [10, 11] but not in C57BL or SV129 mice [12]. A genome-wide linkage analysis using C57BL and ICGN backcross mice carrying TNS2 mutations has suggested significant chronic kidney disease resistance loci on mouse chromosome 2 and 13 [13]. The genetic modifiers and molecular mechanisms leading to the renal phenotypes remain to be established.

In this study, we have clarified the identities of three human TNS2 isoforms and established the loss-of-function effects of TNS2 in cancer cells. These three TNS2 isoforms only differ at their N-termini and these differences do not appear to alter their subcellular localization. Silencing of TNS2 promotes the activation of IRS1, Akt, and Mek, and enhances cancer cell tumorigenicities. Analysis of TNS2 expression patterns in human cancer patients has indicated that TNS2 is rarely overexpressed in cancers and patients with low TNS2 expression are associated with poor relapse-free survival probabilities in breast and lung cancers. These results demonstrate a role of TNS2 in suppressing cell transformation and reduction of tumorigenicity.

## RESULTS

### Three human TNS2 isoforms differ at their N-termini

**Figure 1:**
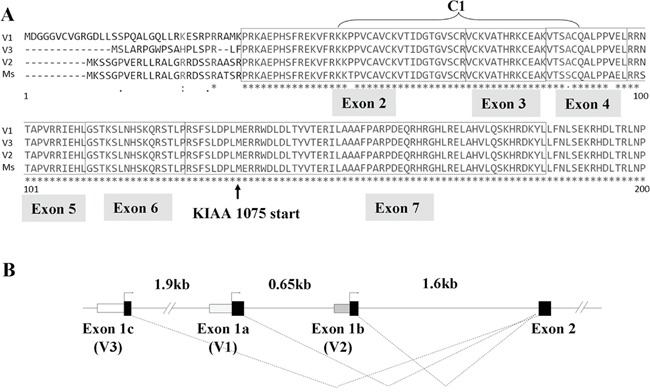
TNS2 has three isoforms in humans **A.** Alignment of the open reading frame encoded by three human isoforms and one mouse transcript. The N-terminal C1 regions and first 7 exons are shown. Identical residues are indicated with * asterisks. Arrow indicates the start site used in the KIAA1075 clone. **B.** Schematic overview of the 5′ end of the TNS2 gene. Exons and introns are depicted as boxes and lines, respectively. The independent splicing of alternative 5′ exons (1a, 1b, and 1c) generates three transcripts with different 5′ end sequences.

Several TNS2 isoforms have been reported in various publications. Identification of human TNS2 was first reported in 2002 [4, 14] and it was also called C1-ten because it contained a C1 domain at its N-terminal region [14], which was not found in other tensin members. Three isoforms named V1 (1419 aa), V2 (1409 aa), and V3 were described later [7, 15]. Both reports stated that V3 (derived from KIAA 1075) was the shortest form with no C1 domain. Nonetheless, with careful analysis of the KIAA1075 cDNA sequence, an open reading frame upstream of the proposed start codon was identified, with this region encoded identical amino acid sequence with those of V1 and V2 including the C1 domain. However, no start codon upstream of the C1 coding region was found in the original KIAA1075. Using 5′-RACE we tested whether a start codon upstream of the C1 coding region could be found. In addition to the sequences coding for the N-termini of V1 and V2, we also identified a new sequence with a start codon leading to a novel N-terminus (Figure 1A). With this new coding N-terminal sequence, this isoform would encode for a 1403 amino acid polypeptide. Given this data, it is very likely that the original KIAA1075 is an incomplete form of the TNS2 V3 isoform. Therefore, all three isoforms encode identical protein sequences except 35, 25, and 19 residues at the very N-terminal of V1, V2, and V3 respectively. Alignment with human genomic sequence allowed us to clarify the positions of exon 1a, 1b, and 1c that contained the unique sequences for V1, V2, and V3, and all three isoforms share the rest of exons (Figure 1B).

### Expression profiles of TNS2 isoforms

**Figure 2:**
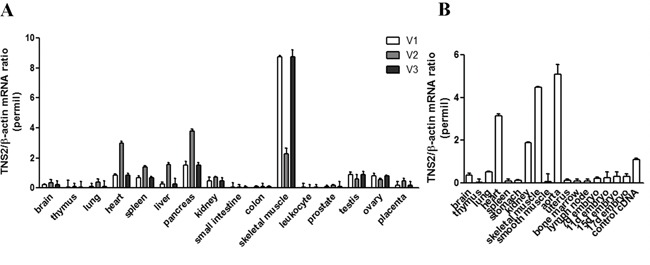
TNS2 expression patterns in humans and mice **A.** Real-time qPCR analysis of *TNS2* gene using three human isoform specific primers (described in M&M) in human tissues. **B.** TNS2 expression in mouse tissues. The relative mRNA values have been normalized to β-actin.

Primers specific for each isoform were used for qPCR assay to establish the expression profiles in human tissues. Higher expression levels of TNS2 V1 and V3 were found in skeletal muscle, and V2 was in pancreas and heart (Figure 2A). Overall, V1 and V3 appeared to have similar tissue expression patterns that were different from V2. Interestingly, only one mouse TNS2 was found in NCBI reference sequence database. Based on its encoded amino acid sequence, mouse TNS2 is more related to human V2 form (Figure 1A). The qPCR result showed that mouse TNS2 mRNA levels were higher in heart, kidney, skeletal muscle and aorta (Figure 2B).

### Subcellular localization of TNS2 isoforms

**Figure 3:**
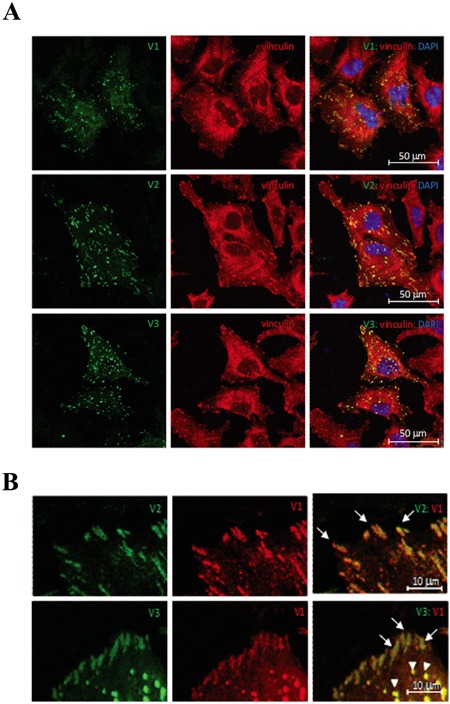
Subcellular localization of TNS2 isoforms **A.** HeLa cells were transfected with EGFP-TNS2 V1, V2, or V3 and stained with anti-vinculin antibodies. TNS2, vinculin, and nuclei are shown in green, red, and blue, respectively. **B.** Tomato-TNS2 V1 was cotransfected with EGFP-TNS2 V2 or V3 into HeLa cells. TNS2 V2 or V3 are shown in green, while TNS2 V1 is in red. Arrows and arrowheads indicate focal adhesions and punctuate staining respectively

To investigate the subcellular localization of these three isoforms, EGFP-tagged TNS2 constructs were transfected into HeLa cells. All three isoforms co-localized with vinculin at focal adhesion sites (Figure 3A). To further distinguish any potential difference in subcellular localization among these isoforms, we generated Tomato-tagged TNS2 V1 and cotransfected with EGFP-tagged V2 or V3 into HeLa cells. TNS2 V1, V2, and V3 isoforms had the same distribution at focal adhesions as well as the punctuate staining (Figure 3B) reported in several publications [15, 16]. These results indicated that the N-terminal variations in TNS2 isoforms had no detectable effect on their subcellular localization.

### Silencing of TNS2 enhances tumorigenicity of cancer cell lines

**Figure 4:**
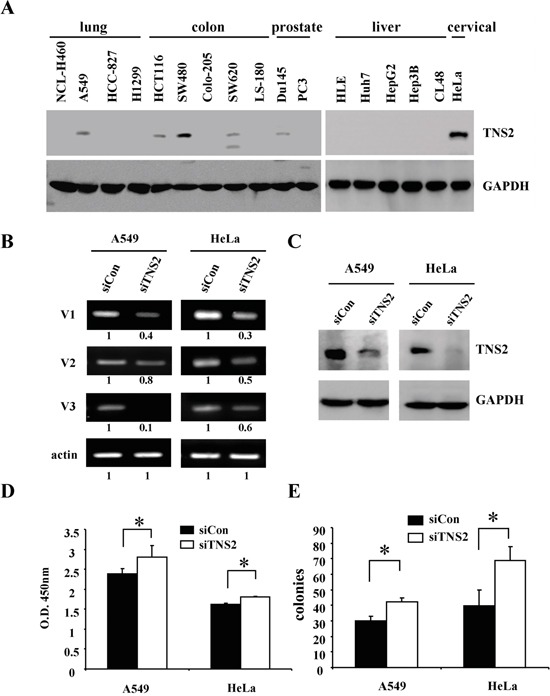
Silencing of TNS2 enhances cancer cell tumorigenicity **A.** Cell lysates from indicated cell lines were blotted with TNS2 or GAPDH antibodies. A549 or HeLa transfected with siRNA against TNS2 (siTNS2) or control (siCon) were analyzed by RT-PCR assays with isoform specific primers **B.,** blotted with TNS2 or GAPDH antibodies **C.,** or analyzed for their 4-day growth rates by WST1 assays **D.** and their colony formation activities **E.** Numbers under RT-PCR gel panels (B)indicated band intensities comparing to corresponding siCon controls.

**Figure 5:**
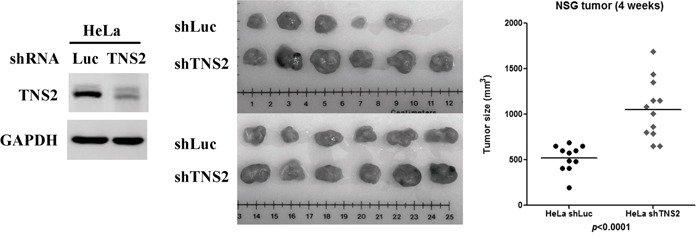
Silencing of TNS2 promotes tumor growth in xenograft models shTNS2 and shLuc in HeLa cells were used for xenograft assay in NSG mice. Down-regulation of TNS2 protein level in shTNS2 was confirmed by western blot analysis (left panel). Tumors are pictured (middle panel) along with the measured average size (right panel). * *P-value* <0.05

**Figure 6:**
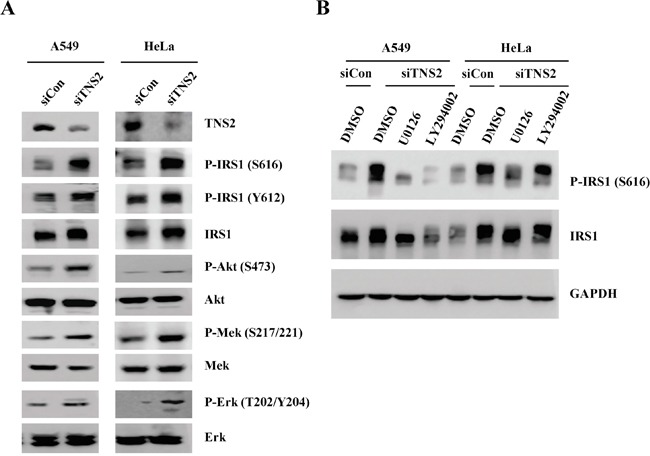
Silencing of TNS2 activates IRS1, AKT, and Mek signaling networks **A.** Cell lysates from A549 or HeLa transfected with siTNS2 or siCon were blotted with indicated antibodies. **B.** Cells treated with inhibitors to Mek (U0126), PI3K (LY294002), or DMSO were subjected to western blot analysis with antibodies against IRS1, P-IRS1(S616), or GAPDH.

A panel of human cancer cell lines was screened by immunoblot analysis to evaluate for TNS2 protein expression. While TNS2 protein was detected in A549, HCT116, SW480, SW620, DU145, and HeLa, no TNS2 band was observed in H460, HCC827, Colo205, LS-180, PC3, HLE, Huh7, HepG2, Hep3B, and CL48 cell lysates (Figure 4A). To establish the functional roles of TNS2 in cancer cells, TNS2 was down-regulated by the siRNA approach in HeLa and A549 cells. We selected HeLa due to its high TNS2 protein level and A549, the only TNS2-positive lung cancer cell line we found, because of the clinical significance of TNS2 in lung cancer (see below). Although the siRNA targeted the common region shared by all three isoforms of TNS2, it appeared to be more efficient in reducing the mRNA level of V1 form, also the dominant form, in A549 and HeLa cells (Figure 4B). Nonetheless, TNS2 protein levels were markedly down-regulated by siRNA against TNS2 (siTNS2) in both cell lines (Figure 4C). Silencing of TNS2 promoted cancer cell proliferation and colony formation activities in both HeLa and A549 cells (Figure 4D&4E). For xenograft assays, a shRNA lentivirus targeting human TNS2 3′ UTR region was used for establishing TNS2 knockdown in HeLa cells. HeLa control (shLuc) and TNS2 knockdown (shTNS2) cells were injected into immune deficient NSG mice. shTNS2 HeLa cells developed significantly larger tumors than shLuc cells (Figure 5), demonstrating a role of TNS2 in suppressing tumorigenicity. To establish the underlying mechanisms, we investigated the potential involvements of several signaling molecules. Silencing of TNS2 markedly increased the activated forms of IRS1 (human pS616, pY612, or mouse pS612, pY608), Akt (pS473), Mek (pS217/221), and Erk (pT202/pY204), as well as IRS1 protein levels (Figure 6A), suggesting that TNS2 negatively regulated these pathways. To examine whether the increased IRS1 protein and phosphorylation levels were dependent on Mek and/or Akt pathways, TNS2 knockdown cells were treated with inhibitors to Mek or PI3K (upstream of Akt). While Mek (U0126) and PI3K (LY294002) inhibitors suppressed IRS1 phosphorylation and protein levels induced by TNS2 knockdown in A549 cells, only U0126 significantly reduced IRS phosphorylation in HeLa cells (Figure 6B), suggesting that the Mek pathway is critical for enhancing IRS1 activation levels in TNS2 knockdown cells.

### TNS2 is down-regulated in human cancers and low expression is associated with poor survival potential

**Figure 7:**
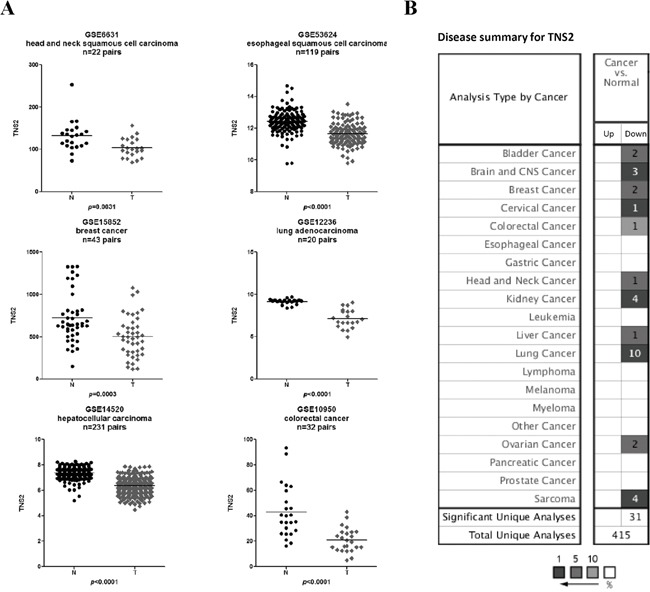
TNS2 expression in human normal and tumor samples **A.** Scatter plot of TNS2 expression values from expression profiles in six independent datasets: 22 pairs of normal and head and neck squamous cell carcinoma; 119 pairs of normal and esophageal squamous cell carcinoma; 43 pairs of normal and breast cancer samples; 20 pairs of normal and lung adenocarcinoma; 231 pairs of normal and hepatocellular carcinoma; and 32 pairs of normal and colorectal cancer. The statistical significances between normal (N) and tumor (T) samples were calculated with a two-tailed unpaired Student's t-test. *P-values* <0.05 were considered to be statistically significant. **B.** The expression between normal and various types of cancers was analyzed through Oncomine with default thresholds. The number of Oncomine profiles with significant TNS2 under-expression for each cancer-type is shown in the box under “Down”. All thirty-one significant cases showed TNS2 down-expression in cancer samples. None of 415 Oncomine profiles showed TNS2 up-regulation. The grey scales indicated TNS2 was on the top 1%, 5% or 10% altered genes. For example, TNS2 expression was among the top 5% genes that were the most down-regulated in 2 bladder cancer profiles in Oncomine.

**Figure 8:**
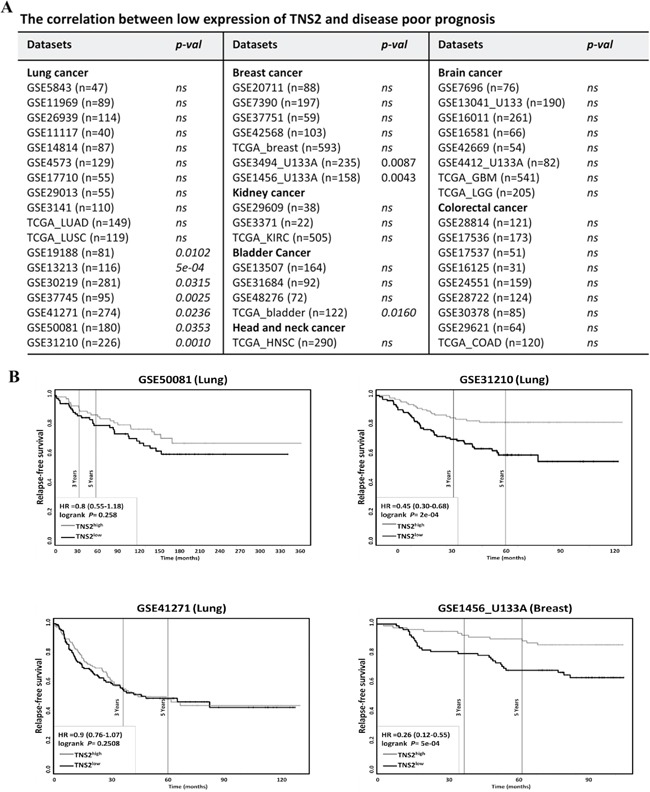
Overall survival analysis of cancer patients according to TNS2 expression levels **A.** The correlation between TNS2 expression and patients' overall survival probability. The patients' survival probabilities of TNS2 expression in lung cancer (18 datasets), breast cancer (7 datasets), kidney cancer (3 datasets), bladder cancer (4 datasets), head and neck cancer (1 dataset), brain cancer (8 datasets), and colorectal cancer (9 datasets) were analyzed by using the online survival analysis tools (PROGgeneV2 and Kaplan Meier-plotter). *P*-values <0.05 are considered statistically significant. *ns*: not significant. **B.** Kaplan-Meier survival curve analyses were conducted in four larger datasets (N>150) with available disease relapse information. One lung cancer (GSE31210) and one breast cancer (GSE1456_U133A) groups showed low TNS2 expression patients associated with poor relapse-free survival probabilities. The log rank *p* value, hazard ratio, lower and upper confidence intervals of hazard ratio retrieved from each cohort was displayed on the survival plot legend.

To determine the clinical relevance of TNS2 expression in cancer patients, we have searched and analyzed publicly available expression databases. Six expression datasets containing human normal and cancer paired (≥ 20) samples from GEO were found and analyzed. When compared to their paired adjacent normal samples, TNS2 mRNA was shown to be significantly downregulated in all six cancer types (Figure 7A), including head and neck squamous cell carcinoma (GSE6631), esophageal squamous cell carcinoma (GSE53624), breast cancer (GSE15852), lung adenocarcinoma (GSE12236), hepatocellular carcinoma (GSE14520), and colorectal carcinoma (GSE10950). In addition, by using the default thresholds set of *p*-value ≤ 10^−4^, 2-fold change and 10% top gene rank, 31 out of a total of 415 profiles in the Oncomine database showed significant TNS2 under-expression (Figure 7B). These included bladder, brain, breast, cervical, colon, head and neck, kidney, liver, lung, ovarian cancers and sarcoma. Furthermore, using online survival analysis tools (PROGgeneV2 and Kaplan Meier-plotter) [17–21], the correlation of low TNS2 expression with poor overall survival probability was analyzed in various cancer cohorts (Figure 7A). Although many databases did not show correlations, these were generally with smaller sample sizes. On the other hand, low expression of TNS2 was significantly associated with poor overall prognosis in seven lung cancer cohorts, two breast cancer cohorts and one bladder cancer group (Figure 8A). These groups usually had larger than 150 patients with more than 5 years follow up. In addition to the overall survival, patients' relapse-free survival probabilities of TNS2 expression in 3 lung cancer datasets (GSE50081, GSE41271, and GSE31210) and 1 breast cancer dataset (GSE1456_U133A) were available and analyzed by using the online survival analysis tools at PROGgeneV2. One lung cancer (GSE3210) and one breast cancer (GSE1456_U133A) datasets showed the significant correlation of low TNS2 expression with poor relapse-free survival (Figure 8B).

## DISCUSSION

In this report, we have clarified the identities of three human TNS2 isoforms. Although it was previously described that only V1 and V2 contained the C1 domain [7, 15], our current studies strongly suggested that all three isoforms shared almost identical sequences, including the C1, PTP, SH2 and PTB domains. The only difference among these isoforms resides at their very N-terminal sequences. However, these sequence varieties do not lead to difference in terms of TNS2 protein subcellular localization. With the exception that V1 and V3 expressional profiles are different from that of V2, the biological significance of these sequence variations is currently not clear.

Our TNS2 knockdown experiments have indicated the role of TNS2 in suppressing tumorigenicity of cancer cells (Figure 5). However, it has been reported that overexpression of TNS2 (KIAA form) promoted the tumorigenicity of BEL7402 and Hep3B [7]. On the other hand, overexpression of TNS2 V2 reduced BEL7402 cancer cell colony formation activity [6]. No effect was observed with TNS2 overexpression in DU145 prostate cancer cells [15]. By analyzing human cancer expression profiles, we found TNS2 was down-regulated in a variety of cancers, including hepatocellular carcinoma (HCC) patients (Figure 7). For comparison, through Oncomine we applied the same thresholds for analysis of the expression of the tumor suppressor PTEN. In a total of 449 profiles, PTEN was over-expressed in 3 profiles and under-expressed in 15 groups. These results indicated that under-expression of TNS2 was more common and consistent than PTEN in various cancer types. In addition, poor relapse-free survival rates were associated with patents with lower TNS2 levels in one lung and one breast cancer cohorts (Figure 8). These findings strongly support that experimental results obtained from silencing studies in cancer cells were more relevant to clinical conditions and that TNS2 overexpression in cancer patients probably is a very rare event. Nonetheless, it has been reported that the original V3 form (1285 aa with no C1 domain) was overexpressed in HCC tumor (in 50 pairs) and was associated with venous invasion (p = 0.037), tumor microsatellite formation (p = 0.033), and nonencapsulation (p = 0.049) [7]. Overexpression of GFP-V3 promoted liver cancer cell proliferation, migration, invasion, colony formation and orthotopic liver xenograft [7]. These findings suggest an oncogenic effect of TNS2 in HCC. The reasons for the discrepancy between their and our results are not clear and require further studies.

IRS1 (insulin receptor substrate 1) was originally identified as a critical substrate of the insulin receptor [22]. In the insulin signaling, IRS1 binds to and is tyrosine phosphorylated by activated insulin receptor. Phosphorylated IRS1 provides docking sites for numerous signaling molecules including Grb2, p85, Nck, Crk, and Fyn, which lead to activation of several downstream pathways, such as MAP kinase and PI3K pathways [23]. IRS1 also mediates signals to promote tumor progression. Overexpression of IRS1 in cells promoted cell transformation and in mice lead to tumor development [24, 25]. Therefore, TNS2 may suppress cell transformation by regulating IRS1 protein and its activation levels. In the absence of TNS2, elevated IRS1 signaling may promote tumorigenicity in cancer cells. This is consistent with recent finding that TNS2 acts as a protein tyrosine phosphatase of IRS1 and dephosphorylation of pY612 of IRS1 by TNS2 accelerates IRS1 degradation [26]. Our studies further suggest that TNS2 may suppress IRS1 S616 phosphorylation through regulating Mek and possibly also Akt activities. How TNS2 can negatively regulate Mek and Akt is under investigation.

## MATERIALS AND METHODS

### TNS2 cDNA expression plasmids

The 5′-end sequence of the TNS2 gene was amplified by using a commercially available 5′ Rapid Amplification of cDNA Ends (5′RACE) kit (Life Technologies) according to the manufacturer's instructions. Briefly, one reverse primer 105R (5′-TAGTACGACCACGTGC TGTGGGTCAG-3′) shared by all three isoforms was designed and subjected to a 5′RACE reaction using human control cDNA. The amplified fragments were subcloned into pCR2.1.TOPO (Invitrogen) and sequenced. On the basis of this sequence information, three non-identical clones were obtained. In order to construct the full length of the TNS2 cDNA isoforms, three different forward primers including an EcoRI site and the first ATG codon were designed as below:

V1: 5′-CGGAATTCATGGATGGGGGTGGAGTATG-3′

V2: 5′-CGGAATTCATGAAGTCCAGCGGCCCTG-3′

V3: 5′-CGGAATTCATGTCCCTGGCGCGGCCCGG-3′

PCR amplification was performed using each forward primer and the reverse primer 105R. The amplified DNA was subcloned into the EcoRI and PmlI sites of GFP-TNS2 (Chen et al., 2002) to generate pEGFPC2-TNS2V1, pEGFPC2-TNS2V2, and pEGFPC2-TNS2V3. pTomato-TNS2V1 was generated by swapping EGFP with tdTomato using NheI and BsrGI sites.

### Antibodies

Rabbit polyclonal anti-TNS2 KIAA was raised as described previously [4]. Anti-IRS1 (#3400), anti-pS616 (mouse S612)-IRS1 (#3203), anti-Mek (#4694), anti-pS217/221- Mek (#9154), anti-Erk (#4695), anti-pT202/Y204-Erk (#4370) were purchased from Cell Signaling Technology. Anti-pY612-IRS1 (#09432) was from Millipore. And anti-GAPDH (#4300) was from Ambion. Akt-pS473 (# 7985) was from Santa Cruz Biotechnology.

### Cell culture, siRNA transfection and lentiviral shRNA infection

HeLa and A549 cell lines were purchased from ATCC and cultured in Eagle's Minimum Essential Medium or Dulbecco's modified Eagle's medium (GIBCO) supplemented with 10 % FBS (Sigma) and 1% penicillin/streptomycin. TRC lentiviral shRNA targeting human TNS2 (TRCN0000272886) was obtained from the National RNAi Core Facility (Taiwan). The TNS2 targeting sequences are TCAGAGCCCACATCAACACTG for shRNA and CAGCAAAGAUCCUCUGGAA for siRNA.

### RNA extraction and RT-qPCR

Total RNA was isolated using TriZol (Invitrogen) reagent per the manufacturer instructions. The cDNA was synthesized from total RNA using the High Capacity cDNA Reverse Transcription kit (Applied Biosystems). Real time PCR was performed using SYBR green master mix in a 7900HT Fast Real-Time PCR System (Applied Biosystems). Primers used for TNS2 quantitative PCR are listed below:

V1 (forward: 5′-GGGTGGAGTATGTGTTGGGA-3′; reverse: 5′-GAAAACCTTCTCCCGGAAGC-3′)

V2 (forward: 5′-ACACCATGAAGTCCAGCGG-3′; reverse: 5′-GAAAACCTTCTCCCGGAAGC-3′)

V3 (forward: 5′-CTCTCTCCCCTCGGCTCTTT-3′; reverse: 5′-GAAAACCTTCTCCCGGAAGC-3′)

The relative expressions levels of TNS2 in the tissues are shown following normalization with β-actin transcript values.

### Western blotting

Cultured cells were lysed with RIPA buffer (50 mM Tris Cl, pH 7.4/150 mM NaCl/5 mM EDTA/1% Nonidet P-40/1% sodium deoxycholate/0.1% SDS) with protease and phosphatase inhibitors (Roche). The protein concentration was determined using the BioRad protein assay Kit (BioRad). Lysates were separated in a 7-15 % sodium dodecyl sulfate–polyacrylamide electrophoresis (SDS-PAGE) gel, transferred to nitrocellulose membranes, and incubated with indicated antibodies and visualized by enhanced chemiluminescence (ECL).

### Immunofluorescence and confocal microscopy

Cells cultured on acid-treated coverslips were fixed with 4 % paraformaldehyde/PBS for 15 min and subsequently permeabilized with 0.1 % Triton X-100/PBS for 5 min. After blocking with 5 % goat serum, the cells were incubated with primary antibodies, anti-vinculin (1:200) followed by appropriate secondary antibodies conjugated with Alexa Flour (Invitrogen). The coverslips were mounted on slides using DAPI-containing mounting solution (Vector Laboratories) and digital images were acquired with a Zeiss LSM 710 confocal system.

### Proliferation and colony formation assays

Cells were transfected with control siRNA or TNS2 siRNA (sigma) using Lipofectamine 2000 (Invitrogen). After 48h, 10,000 cells were seeded into a 48 well plate and cultured for 4 days. The growth of cells was measured using the WST1 cell proliferation assay system (Roche) according to manufacturer instructions. For colony formation assay, cells were co-transfected with EGFPC2 plasmid and control siRNA or TNS2 siRNA. After 48h, 5,000 cells were seeded and selected by 400 ug/ml Geneticin (Invitrogen) in a 6 well plate. After 2 weeks of selection, colonies were fixed with formaldehyde and stained with crystal violet.

### Xenograft models

Cells (2 × 10^6^) were suspended in 200 μL of 50 % matrigel in PBS and injected subcutaneously into the both flanks of NSG mice. After 4 weeks, mice were killed, and tumors removed for analysis. Tumor diameters were measured with digital calipers, and the tumor volume in mm^3^ was calculated by the formula, volume = (width)^2^ x length/2. All animal procedures were approved by the Institutional Animal Care and Use Committee (IACUC) at the University of California Davis.

### Microarray database analyses

The expression between normal and various types of cancers was analyzed by Oncomine. Default threshold *p*-value ≤ 10^−4^, 2-fold change, and 10% gene rank set was used to generate the disease summary of TNS2. Gene expression data from Gene Expression Omnibus (GEO) database NCBI was downloaded to analyze the TNS2 expression in normal and cancer paired samples. Selection criteria for all publicly available datasets required each dataset to include normal and cancer paired samples for more than twenty patients. Six microarray datasets containing the normal and tumor pair samples were selected from the NCBI GEO database, including GSE6631 (22 pairs of head and neck squamous cell carcinoma), GSE53624 (119 pairs of esophageal squamous cell carcinoma), GSE15852 (43 pairs of breast cancer), GSE12236 (20 pairs of lung adenocarcinoma), GSE14520 (231 pairs of hepatocellular carcinoma), and GSE10950 (32 pairs of colorectal cancer). Significantly expressed TNS2 gene is determined based on the comparison of the normal and tumor data. Scattered plot of TNS2 gene expression value (y-axis) for each cancer subtype was generated by GraphPad Prism 5. The patients' survival probabilities of TNS2 expression in various cancers were analyzed by using the online survival analysis tools and datasets at www.watson.compbio.iupul.edu for PROGgeneV2 and www.kmplot.com for Kaplan Meier-plotter. Each cohort was divided into two subgroups based on median expression of TNS2 to analyze the correlation between TNS2 expression and overall survival. The Kaplan-Meier plots, log rank p value, hazard ratio, lower and upper confidence intervals of hazard ratio displayed on the survival plot legend were also generated using these online tools.

### Statistical analysis

Statistical analysis was performed using the using GraphPad Prism 5. All results are expressed as mean ± SD. Significance was defined as *p* ≤0.05.

## References

[1] Winograd-Katz SE, Fassler R, Geiger B, Legate KR (2014). The integrin adhesome: from genes and proteins to human disease. Nat Rev Mol Cell Biol.

[2] Lo SH (2004). Tensin. The international journal of biochemistry & cell biology.

[3] Hafizi S, Ibraimi F, Dahlback B (2005). C1-TEN is a negative regulator of the Akt/PKB signal transduction pathway and inhibits cell survival, proliferation, and migration. FASEB J.

[4] Chen H, Duncan IC, Bozorgchami H, Lo SH (2002). Tensin1 and a previously undocumented family member, tensin2, positively regulate cell migration. Proc Natl Acad Sci U S A.

[5] Clark K, Howe JD, Pullar CE, Green JA, Artym VV, Yamada KM, Critchley DR (2010). Tensin 2 modulates cell contractility in 3D collagen gels through the RhoGAP DLC1. Journal of cellular biochemistry.

[6] Yam JW, Ko FC, Chan CY, Jin DY, Ng IO (2006). Interaction of deleted in liver cancer 1 with tensin2 in caveolae and implications in tumor suppression. Cancer Res.

[7] Yam JW, Ko FC, Chan CY, Yau TO, Tung EK, Leung TH, Jin DY, Ng IO (2006). Tensin2 variant 3 is associated with aggressive tumor behavior in human hepatocellular carcinoma. Hepatology.

[8] Cho AR, Uchio-Yamada K, Torigai T, Miyamoto T, Miyoshi I, Matsuda J, Kurosawa T, Kon Y, Asano A, Sasaki N, Agui T (2006). Deficiency of the tensin2 gene in the ICGN mouse: an animal model for congenital nephrotic syndrome. Mammalian genome.

[9] Kato T, Mizuno S, Taketo MM, Kurosawa TM (2008). The possible involvement of tensin2 in the expression and extension of nephrin by glomerular podocytes in mice. Biomed Res.

[10] Uchio-Yamada K, Sawada K, Tamura K, Katayama S, Monobe Y, Yamamoto Y, Ogura A, Manabe N (2013). Tenc1-Deficient Mice Develop Glomerular Disease in a Strain-Specific Manner. Nephron Experimental nephrology.

[11] Uchio-Yamada K, Monobe Y, Akagi KI, Yamamoto Y, Ogura A, Manabe N (2016). Tensin2-deficient mice on FVB/N background develop severe glomerular disease. The Journal of veterinary medical science.

[12] Nishino T, Sasaki N, Nagasaki K, Ichii O, Kon Y, Agui T (2012). The 129 genetic background affects susceptibility to glomerulosclerosis in tensin2-deficient mice. Biomed Res.

[13] Sasaki H, Sasaki N, Nishino T, Nagasaki K, Kitamura H, Torigoe D, Agui T (2014). Quantitative trait Loci for resistance to the congenital nephropathy in tensin 2-deficient mice. PLoS One.

[14] Hafizi S, Alindri F, Karlsson R, Dahlback B (2002). Interaction of Axl receptor tyrosine kinase with C1-TEN, a novel C1 domain-containing protein with homology to tensin. Biochem Biophys Res Commun.

[15] Hafizi S, Sernstad E, Swinny JD, Gomez MF, Dahlback B (2010). Individual domains of Tensin2 exhibit distinct subcellular localisations and migratory effects. The international journal of biochemistry & cell biology.

[16] Moon KD, Zhang X, Zhou Q, Geahlen RL (2012). The protein-tyrosine kinase Syk interacts with the C-terminal region of tensin2. Biochim Biophys Acta.

[17] Goswami CP, Nakshatri H (2013). PROGgene: gene expression based survival analysis web application for multiple cancers. J Clin Bioinforma.

[18] Goswami CP, Nakshatri H (2014). PROGgeneV2: enhancements on the existing database. BMC Cancer.

[19] Gyorffy B, Benke Z, Lanczky A, Balazs B, Szallasi Z, Timar J, Schafer R (2012). RecurrenceOnline: an online analysis tool to determine breast cancer recurrence and hormone receptor status using microarray data. Breast Cancer Res Treat.

[20] Gyorffy B, Lanczky A, Eklund AC, Denkert C, Budczies J, Li Q, Szallasi Z (2010). An online survival analysis tool to rapidly assess the effect of 22,277 genes on breast cancer prognosis using microarray data of 1,809 patients. Breast cancer research and treatment.

[21] Gyorffy B, Surowiak P, Budczies J, Lanczky A (2013). Online survival analysis software to assess the prognostic value of biomarkers using transcriptomic data in non-small-cell lung cancer. PLoS One.

[22] White MF, Maron R, Kahn CR (1985). Insulin rapidly stimulates tyrosine phosphorylation of a Mr-185,000 protein in intact cells. Nature.

[23] Gual P, Le Marchand-Brustel Y, Tanti JF (2005). Positive and negative regulation of insulin signaling through IRS-1 phosphorylation. Biochimie.

[24] Dearth RK, Cui X, Kim HJ, Hadsell DL, Lee AV (2007). Oncogenic transformation by the signaling adaptor proteins insulin receptor substrate (IRS)-1 and IRS-2. Cell cycle.

[25] Gibson SL, Ma Z, Shaw LM (2007). Divergent roles for IRS-1 and IRS-2 in breast cancer metastasis. Cell cycle.

[26] Koh A, Lee MN, Yang YR, Jeong H, Ghim J, Noh J, Kim J, Ryu D, Park S, Song P, Koo SH, Leslie NR, Berggren PO, Choi JH, Suh PG, Ryu SH (2013). C1-Ten is a protein tyrosine phosphatase of insulin receptor substrate 1 (IRS-1), regulating IRS-1 stability and muscle atrophy. Molecular and cellular biology.

